# Comparative activity of sulbactam and sulbactam/durlobactam against carbapenem-resistant *A. baumannii* isolates producing OXA-23 or OXA-23 plus PER-1 enzymes

**DOI:** 10.1038/s41429-026-00919-x

**Published:** 2026-04-10

**Authors:** Hasan Cenk Mirza, Aylin Üsküdar Güçlü, Sezin Ünlü, Süleyman Yalçın

**Affiliations:** 1 Department of Medical Microbiology, Başkent University Faculty of Medicine, Ankara, Türkiye; 2 Ministry of Health, General Directorate of Public Health, Microbiology Reference Laboratory, Ankara, Türkiye

**Keywords:** Antibiotics, Bacteriology

## Abstract

Our objective was to compare the in vitro activity of sulbactam and sulbactam/durlobactam against carbapenem-resistant *A. baumannii* isolates carrying *bla*_OXA−23_ or both *bla*_OXA−23_ and *bla*_PER-1_ genes. We also aimed to investigate the possible mechanisms of resistance in sulbactam/durlobactam resistant isolates. Twenty-four carbapenem-resistant *A. baumannii* isolates (12 carrying *bla*_OXA−23_, 12 carrying *bla*_OXA−23_ and *bla*_PER-1_) were included in the study. Genetic relations among isolates were determined by Pulsed-Field Gel Electrophoresis (PFGE). MICs of sulbactam and sulbactam/durlobactam were determined by gradient diffusion method. Whole-genome sequencing was performed on sulbactam/durlobactam resistant isolates. Among 24 isolates, PFGE revealed 5 distinct major clusters. One (4.2%) and 22 (91.7%) of isolates were susceptible to sulbactam and sulbactam/durlobactam, respectively. Sulbactam MIC_50_ value for isolates carrying *bla*_OXA−23_ plus *bla*_PER-1_ (32 mg/L) were 2.7 fold higher than that for isolates carrying *bla*_OXA−23_ (12 mg/L). However, similar sulbactam/durlobactam MIC_50_ values were observed for isolates carrying *bla*_OXA−23_ plus *bla*_PER-1_ (1 mg/L) or *bla*_OXA−23_ (1.5 mg/L). Both the two sulbactam/durlobactam resistant isolates had mutations in PBP3 gene. The isolate with sulbactam/durlobactam MIC of 12 mg/L had A515V and C1546T mutations in PBP3 gene. Also, the AdeN (repressor of AdeIJK efflux system) was absent in this isolate. The isolate with sulbactam/durlobactam MIC of >64 mg/L had one amino acid insertion at position 374 (-374D) in PBP3. Sulbactam/durlobactam demonstrated high activity against carbapenem-resistant *A. baumannii* isolates. Sulbactam/durlobactam resistance was likely due to mutations in PBP3 gene. Overexpression of AdeIJK efflux system may also have contributed to resistance in one isolate.

## Introduction

In May 2024, the World Health Organization (WHO) updated its list of antibiotic-resistant bacteria which pose the greatest threat to human health. Carbapenem-resistant *Acinetobacter baumannii* is still included in the critical priority category of list [1]. Most of the antibiotics are ineffective against infections caused by carbapenem-resistant *A. baumannii*, emphasizing the need for development of new antibiotics against this opportunistic pathogen [2].

The main mechanism responsible for carbapenem resistance in *A. baumannii* is the production of class D β-lactamases; primarily the OXA-23, OXA-58 and OXA-24/40 [3]. Resistance in *A. baumannii* to broad-spectrum cephalosporins is predominantly due to the overexpression of the AmpC β-lactamases or production of extended-spectrum β-lactamases (ESBL) [4]. PER-1 β-lactamase is an ESBL enzyme belong to Ambler class A and was first detected in a *Pseudomonas aeruginosa* isolated from a Turkish patient in France in 1993. Later, PER enzymes were also reported in *Acinetobacter* isolates from different parts of the world including Türkiye, South Korea, France, Belgium, China, India, Iran, Saudi Arabia, Tunisia and Argentina [5, 6].

Sulbactam is a first-generation β-lactamase inhibitor which has limited activity against Ambler class A serine β-lactamases [3]. In addition, sulbactam has intrinsic activity against *A. baumannii*, due to the inhibiton of cell wall synthesis by binding to penicillin-binding proteins (PBPs) including PBP1a, PBP1b and PBP3 [7, 8]. However, susceptibility of sulbactam to degradation by various β-lactamases found in many *Acinetobacter* spp. isolates limits its use in infections caused by this microorganism [9].

Durlobactam is a novel non-β-lactam β-lactamase inhibitor which inhibits Ambler class A, C and D β-lactamases [10, 11]. In 2023, durlobactam in combination with sulbactam was approved by the US Food and Drug Administration (FDA) for the treatment of hospital-acquired bacterial pneumonia and ventilator-associated bacterial pneumonia caused by *Acinetobacter baumannii-calcoaceticus* complex [12]. The sulbactam/durlobactam zone diameter and MIC breakpoints assigned by FDA were also approved by CLSI in 2023 and were published in the 34th edition of CLSI M100 document in 2024 [13].

Our objective was to investigate the in vitro activity of sulbactam and sulbactam/durlobactam against a collection of carbapenem-resistant *A. baumannii* isolates including strains carrying *bla*_OXA−23_ gene or carrying both *bla*_OXA−23_ and *bla*_PER-1_ genes. We also aimed to investigate the mechanisms responsible for sulbactam/durlobactam resistance among *A. baumannii* isolates.

## Materials and methods

This study was approved by Başkent University Institutional Review Board (Project no: DA24/07) and supported by Başkent University Research Fund.

### Bacterial isolates

A total of 24 clinical isolates of carbapenem-resistant *A. baumannii* including strains carrying *bla*_OXA−23_ (*n* = 12) or carrying both *bla*_OXA−23_ and *bla*_PER-1_ (*n* = 12) were included in the study. Strains were randomly selected from a collection of *A. baumannii* clinical isolates those were characterized in our previous study and stored at Medical Microbiology Laboratory of Başkent University Medical Faculty [14]. Strains were isolated in 2020 and 2021. One isolate per patient was included. Isolates were obtained from blood (*n* = 12), tracheal aspirate (*n* = 11), and bronchoalveolar lavage (*n* = 1).

### Antimicrobial susceptibility testing

MICs of sulbactam and sulbactam/durlobactam were determined with gradient diffusion method using sulbactam MIC test strips (Liofilchem, Roseto degli Abruzzi, Italy) and sulbactam/durlobactam Etest strips (bioMérieux; Marcy-l'Étoile, France). Sulbactam/durlobactam Etest strips were provided by International Health Management Associates (IHMA) within the scope of the Antimicrobial Voluntary Evaluation Program (AVEP). To determine the MICs for isolates, the bacterial suspension equal to the turbidity of 0.5 McFarland standard was swabbed onto Mueller–Hinton agar (Condalab, Madrid, Spain) and allowed to dry. Afterwards, strips containing sulbactam and sulbactam/durlobactam were placed on the surface of agar and the plates were incubated at 35 °C for 16–20 h.

CLSI and EUCAST have not provided sulbactam MIC breakpoints for *Acinetobacter* spp. yet. However, CLSI provides ampicillin/sulbactam MIC breakpoints (susceptible ≤8/4 mg/L, intermediate 16/8 mg/L and resistant ≥32/16 mg/L) for *Acinetobacter* spp. As the sulbactam comprises the active component of the combination, sulbactam MIC breakpoints of ≤4 mg/L (susceptible), 8 mg/L (intermediate) and ≥16 mg/L (resistant) were used [8, 15]. Sulbactam/durlobactam MICs were interpreted according to CLSI criteria in M100-S34 (susceptible ≤4/4 mg/L, intermediate 8/4 mg/L and resistant ≥16/4 mg/L) [15]. *A. baumannii* NCTC 13304 was used as quality control strain.

### Pulsed-field gel electrophoresis (PFGE)

Genetic relations among carbapenem-resistant *A. baumannii* clinical isolates were determined by PFGE according to the protocol described by Gouby et al. [16]. Genomic DNA was lysed with lysis solutions and digested with ApaI restriction enzyme (Takara, Japan). Electrophoresis was performed in a pulsed-field electrophoresis system (CHEF-DR III System; Bio-Rad, USA). Cluster analysis was performed using BioNumerics software. Isolates were defined as closely related if they differ by ≤3 bands and possibly related if they differ by 4–6 bands, according to criteria of Tenover et al. [17].

### Whole-genome sequencing and analysis

To understand the molecular drivers of resistance against sulbactam/durlobactam, all sulbactam/durlobactam resistant isolates were subjected to whole-genome sequencing (WGS) and analyzed for β -lactamase gene content, and variations in PBPs and AdeIJK efflux system. Variations in the AdeIJK efflux system was reported to play role in sulbactam/durlobactam resistance in some *Acinetobacter* spp. isolates [9]. Whole-genome sequencing of a sulbactam/durlobactam susceptible isolate was also performed for comparison.

Genomic DNA extraction of bacterial isolates were performed by using EZ1 automated sample purification system (Qiagen, Germany). DNA was quantified by Qubit 4 fluorometer. The whole genome was sequenced by Oxford Nanopore prometION sequencing system (ONT, Oxford, UK). Libraries were prepared using the Native Barcoding Kit. De novo assemblies of the genomes were generated using flye v. 2.9.3. For the quality of the genome assemblies and trimming, Fastqc v.0.12.1 and Porechop v.0.2.4 were used [18, 19]. The assembled genomes were annotated with RasTK [20] and PROKKA [21]. According to functional gene annotation; PBP1a, PBP1b, PBP3 and efflux pump regions were alligned with the A. *baumannii* ATCC 17978 (GenBank accession number CP018664.1) to detect the mutations by using MEGAX [22]. The existence of β-lactamese encoding genes in the genome was investigated via ResFinder 4.0 [23].

### Statistical analysis

The Mann-Whitney U Test was used to compare sulbactam MICs for ‘*bla*_OXA−23_ carrying’ and ‘*bla*_OXA−23_+*bla*_PER-1_ carrying’ isolates. The same test was also used to compare sulbactam/durlobactam MICs for ‘*bla*_OXA−23_ carrying’ and ‘*bla*_OXA−23_+*bla*_PER-1_ carrying’ isolates. The Wilcoxon Signed-Rank Test was used to compare sulbactam and sulbactam/durlobactam MICs for *A. baumannii* isolates. *P* values of <0.05 were considered statistically significant.

## Results

**Fig. 1:**
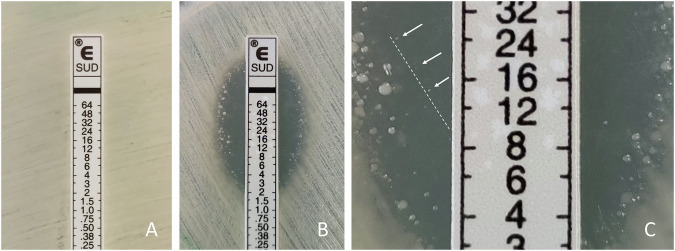
Gradient diffusion test results for two sulbactam/durlobactam-resistant isolates. **A** No zone of inhibiton was observed for the isolate Ab19 which indicates high level (MIC > 64 mg/L) sulbactam/durlobactam resistance. **B** Isolate Ab92 exhibited some small colonies within the inhibiton zone and categorized as resistant to sulbactam/durlobactam. **C** Magnified appearance of (**B**). Isolate Ab92 had sulbactam/durlobactam MIC of 12 mg/L (resistant)

Among 24 carbapenem-resistant *A. baumannii* isolates, only one (4.2%) was susceptible to sulbactam. However, 22 (91.7%) of 24 isolates were susceptible to sulbactam/durlobactam (Table 1). Adding durlobactam to sulbactam restored sulbactam susceptibility in 21 (91.3%) of 23 sulbactam-non-susceptible isolates. The two sulbactam/durlobactam resistant isolates had sulbactam/durlobactam MICs of >64 mg/L and 12 mg/L, respectively (Fig. 1). The isolate with high level resistance to sulbactam/durlobactam (MIC > 64 mg/L) was also highly resistant (MIC > 256 mg/L) to sulbactam. The isolate with sulbactam/durlobactam MIC of 12 mg/L had sulbactam MIC of 24 mg/L. Two sulbactam/durlobactam resistant isolates were positive for *bla*_OXA−23_ but negative for *bla*_PER-1_ (Table 2).

**Table 1:** Sulbactam and sulbactam/durlobactam susceptibilities of isolates carrying *bla*_OXA−23_ or both *bla*_OXA−23_ and *bla*_PER-1_ genes

β-lactamase genes	Sulbactam				Sulbactam/Durlobactam			
	MIC (mg/L)			%S/%I/%R	MIC (mg/L)			%S/%I/%R
	50%	90%	Range		50%	90%	Range	
*bla*_OXA−23_ (*n* = 12)	12	32	4–>256	8.3/16.7/75.0	1.5	12	0.75–>64	83.3/0/16.7
*bla*_OXA−23_ + *bla*_PER-1_ (*n* = 12) (*n* = 12)	32	192	24–>256	0/0/100	1	2	0.75–2	100/0/0
Total	24	192	4–>256	4.2/8.3/87.5	1,5	2	0.75–>64	91.7/0/8.3

**Table 2:** Sulbactam, sulbactam/durlobactam MIC values and sources of 24 *A. baumannii* isolates, and demographic information of patients

Isolate no	Patient age/gender	Ward	Source	β-lactamases	Sulbactam		Sulbactam/durlobactam	
					MIC (mg/L)	Category	MIC (mg/L)	Category
Ab6	78/M	ICU	Tracheal aspirate	OXA-23	4	S	1.5	S
Ab8	80/M	ICU	BAL	OXA-23	12	R	0.75	S
Ab19	75/F	ICU	Tracheal aspirate	OXA-23	>256	R	>64	R
Ab21	79/M	ICU	Blood	OXA-23	12	R	2	S
Ab29	61/F	OG	Blood	OXA-23	12	R	1	S
Ab32	75/M	TS	Tracheal aspirate	OXA-23	12	R	1	S
Ab41	94/F	CD	Blood	OXA-23	32	R	1.5	S
Ab44	67/M	Neurosurgery	Tracheal aspirate	OXA-23	8	I	2	S
Ab49	62/M	GS	Blood	OXA-23	24	R	1.5	S
Ab55	76/F	ICU	Tracheal aspirate	OXA-23	6	I	2	S
Ab70	73/F	ICU	Blood	OXA-23	12	R	2	S
Ab92	45/F	ICU	Blood	OXA-23	24	R	12	R
Ab1	53/M	ICU	Blood	OXA-23 + PER-1	48	R	1.5	S
Ab10	47/M	ICU	Blood	OXA-23 + PER-1	192	R	2	S
Ab15	25/M	BCU	Blood	OXA-23 + PER-1	32	R	1	S
Ab27	68/M	ICU	Tracheal aspirate	OXA-23 + PER-1	64	R	0.75	S
Ab28	28/F	ICU	Blood	OXA-23 + PER-1	64	R	1	S
Ab36	81/M	ICU	Tracheal aspirate	OXA-23 + PER-1	>256	R	2	S
Ab38	65/M	ICU	Tracheal aspirate	OXA-23 + PER-1	24	R	1.5	S
Ab46	87/F	Rheumatology	Blood	OXA-23 + PER-1	24	R	1	S
Ab51	57/M	ICU	Tracheal aspirate	OXA-23 + PER-1	32	R	1.5	S
Ab58	4/M	CVS	Tracheal aspirate	OXA-23 + PER-1	32	R	0.75	S
Ab78	79/F	Neurosurgery	Blood	OXA-23 + PER-1	24	R	1	S
Ab90	4 months/M	CVS	Tracheal aspirate	OXA-23 + PER-1	24	R	1	S

*BAL* bronchoalveolar lavage, *BCU* burn care unit, *CD* chest diseases, *CVS* cardiovascular surgery, *F* female, *GS* general surgery, *ICU* intensive care unit, *I* intermediate, *M* male, *OG* obstetrics and gynecology, *R* resistant, *S* susceptible, *TS* thoracic surgery

Among 24 carbapenem-resistant isolates, 3 were also found to be resistant to colistin with commercial broth microdilution plate (Sensititre FRCOL plate, Thermo Fisher Scientific, USA). However, two sulbactam/durlobactam resistant isolates were susceptible to colistin.

The sulbactam MICs for isolates carrying *bla*_OXA−23_+*bla*_PER-1_ were significantly higher than those for the isolates carrying *bla*_OXA−23_ (*p* = 0.003). However no statistically significant difference was found between sulbactam/durlobactam MICs for isolates carrying *bla*_OXA−23_+*bla*_PER-1_ and isolates carrying *bla*_OXA−23_ (*p* = 0.061). The sulbactam MIC_50_ value for isolates carrying *bla*_OXA−23_ plus *bla*_PER-1_ (32 mg/L) were 2.7 fold higher than that for the isolates carrying *bla*_OXA−23_ (12 mg/L). However, similar sulbactam/durlobactam MIC_50_ values were observed for the isolates carrying *bla*_OXA−23_ plus *bla*_PER-1_ (1 mg/L) and the isolates carrying *bla*_OXA−23_ (1.5 mg/L) (Table 1). Considering a total of 24 isolates, the addition of durlobactam decreased the sulbactam MIC_50_ value by 16-fold (from 24 to 1.5 mg/L) and sulbactam MIC_90_ value by 96-fold (from 192 to 2 mg/L) (Table 1). The sulbactam/durlobactam MICs were significantly lower than sulbactam MICs (*p* < 0.001).

**Fig. 2:**
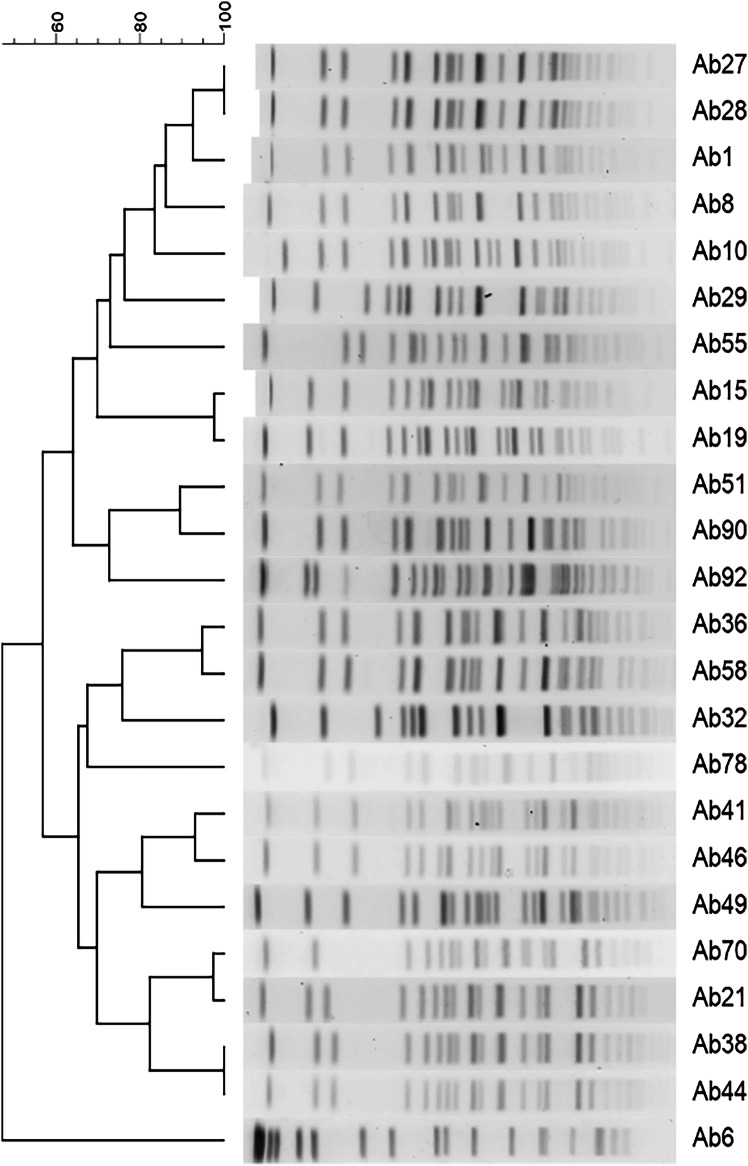
Dendrogram of pulsed-field gel electrophoresis (PFGE) cluster analysis of 24 *A. baumannii* isolates from different patients. Five clusters were found according to banding patterns of each isolate

Among 24 isolates, PFGE revealed 5 distinct major clusters. The largest cluster contained 9 isolates, while the second and third largest clusters had 4 and 7 isolates, respectively. The smaller cluster contained 3 isolates. One isolate (Ab6) had a unique banding pattern compared to the other isolates, forming a distinct, unrelated lineage (Fig. 2).

To understand the molecular mechanisms responsible for sulbactam/durlobactam resistance, two sulbactam/durlobactam-resistant isolates were subjected to whole-genome sequencing. In addition, one randomly selected sulbactam/durlobactam susceptible isolate was subjected to whole-genome sequencing for comparison.

All the three isolates (two sulbactam/durlobactam resistant and one suceptible) carried inherent beta-lactamase genes for *A. baumannii*, *bla*_OXA-66_(*bla*_OXA-51-like_ gene) and *bla*_ADC_. In addition, whole-genome sequencing analysis showed the presence of *bla*_TEM_ in one of the sulbactam/durlobactam resistant isolates.

In both of the two sulbactam/durlobactam resistant isolates, mutations were observed in the genes encoding PBP3. The isolate with sulbactam/durlobactam MIC of 12 mg/L had A515V and C1546T mutations in the PBP3 gene. The isolate with sulbactam/durlobactam MIC of >64 mg/L had one amino acid insertion at position 374 (-374D) in PBP3. In addition to these mutations, both sulbactam/durlobactam resistant isolates encoded three silent mutations (C780T, A906G and A1568T) in PBP3 gene. In sulbactam/durlobactam resistant strains, no mutations were detected in the genes encoding PBP1a and PBP1b, the other targets of sulbactam.

Regarding efflux systems, the AdeN (the repressor of AdeIJK efflux system) was absent in the isolate with sulbactam/durlobactam MIC of 12 mg/L (Table 3).

**Table 3:** Analysis of penicillin-binding proteins, β-lactamase genes and AdeIJK efflux system in sulbactam/durlobactam resistant (*n* = 2) and susceptible (*n* = 1) isolates after whole-genome sequencing

	Isolates		
	Ab92	Ab19	Ab41
Sulbactam/durlobactam MIC (mg/L)	12 (R)	>64 (R)	1.5 (S)
Genbank accession number	PQ382893	PQ382891	PQ382892
Sequence type	218, 2164	1697, 2535	502
Amino acid sequence length of PBP3	610	611	610
PBP3	A515V C1546T *Silent mutations:* C780T A906G A1568T	-374D (one amino acid insertion at position 374) *Silent mutations:* C780T A906G A1568T	V595L
PBP1a	Identical	Identical	Identical
PBP1b	Identical	Identical	Identical
AdeIJK efflux system	AdeN was absent	No mutation	No mutation
β-lactamase genes	*bla*_OXA-23_, *bla*_TEM-1_, *bla*_OXA-66_, *bla*_ADC-73_	*bla*_OXA-23_, *bla*_OXA-66_, *bla*_ADC-30_	*bla*_OXA-23_, *bla*_PER-1_, *bla*_OXA-66_*, bla*_ADC-25_

*R* resistant, *S* susceptible

## Discussion

To our knowledge, this is the first study to evaluate the activity of sulbactam/durlobactam against *A. baumanni* isolates in Türkiye. In this study, the in vitro activity of sulbactam/durlobactam was also compared with that of sulbactam, and the possible mechanisms of resistance in sulbactam/durlobactam resistant isolates were investigated.

The sulbactam MICs for isolates carrying *bla*_OXA−23_ plus *bla*_PER-1_ were higher than those observed for isolates carrying *bla*_OXA−23_ (Table 2). Sulbactam is not an inhibitor of OXA-23 [24]. Instead, it is susceptible to hydrolysis by OXA-23 enzyme, as sulbactam is a β-lactam itself [25]. On the other hand, sulbactam is known to be both a substrate and inhibitor of serine β-lactamases [25] including the ESBL enzyme PER-1 [26].

It was reported that many sulbactam molecules are needed to inactivate a single molecule of a serine β-lactamase. For example, the TEM-1 β-lactamase first hydrolyses ~7000 molecules of sulbactam and then sulbactam functions as an inhibitor of enzyme [27]. Although we could not find a data regarding the number of sulbactam molecules to inactivate PER-1 enzyme, it is likely that large quantities of sulbactam molecules are used for the inactivation of PER-1. Thus, in isolates carrying *bla*_OXA−23_ plus *bla*_PER-1_, lower number of free sulbactam molecules available for the inhibition of PBPs may be the cause of increased sulbactam MICs when compared with isolates that do not carry *bla*_PER-1_.

In a systematic review, the molecular epidemiology of carbapenem-resistant *A. baumannii* isolates in Türkiye was evaluated by analyzing original research articles published between January 2010 and January 2022 [28]. The incidence rates of carbapenemase genes *bla*_OXA−23_ and *bla*_OXA−58_ in carbapenem-resistant *A. baumannii* isolates were reported as 76.4% and 8.4%, respectively. Also, the prevalences of both these genes increased with time. The average positivity rates of *bla*_NDM_ and the ESBL gene *bla*_PER-1_ was reported as 0.1% and 9.7%, respectively [28]. The strains in our study was isolated in 2020 and 2021. The prevalence of OXA-type carbapenemases in *A. baumannii* may have increased from 2020 to 2026. Despite a possible increase in the prevalence of OXA-type carbapenemases, sulbactam/durlobactam may still show high activity against carbapenem-resistant *A. baumannii* isolates as durlobactam is an inhibitor of Ambler class D β-lactamases. However, it is unknown whether the PBP3 mutation patterns or the prevalence of isolates with PBP3 mutations have changed in Türkiye over time. Changes regarding PBP3 may directly influence the activity of sulbactam/durlobactam and may limit the generalizability of our findings from earlier isolates.

In sulbactam/durlobactam combination, sulbactam is known to be the active component against *Acinetobacter* spp. Durlobactam is reported to have no intrinsic activity against *A. baumannii* [11]. Durlobactam protects sulbactam from hydrolysis by β-lactamase enzymes. However it does not inhibit metallo-β-lactamases. For the two sulbactam/durlobactam resistant isolates in our study, β-lactamases likely had very limited effect on sulbactam and sulbactam/durlobactam resistance and other factors were present; because adding durlobactam to sulbactam did not restore sulbactam susceptibility and these isolates did not carry metallo-β-lactamase genes. In one of the isolates, the addition of durlobactam led to a very limited reduction in sulbactam MIC value (from 24 to 12 mg/L). The isolate with high level sulbactam resistance (MIC > 256 MIC mg/L) still exhibited high level resistance (MIC > 64 mg/L) after the addition of durlobactam. In both of the isolates, mutations in PBP3 gene were detected. The first isolate encoded the A515V variant of PBP3 gene. A515V substitution was reported to be associated with sulbactam/durlobactam resistance [7]. However, some authors suggest that A515V substitution is not sufficient to confer sulbactam/durlobactam resistance and additional factors are probably needed [9]. The first isolate had an additional mutation (C1546T) in PBP3 gene. Furthermore, the AdeN (the repressor of AdeIJK efflux system) was absent in this isolate. Durlobactam was reported to be a substrate for the AdeIJK efflux system [9]. The overexpression of this efflux system and the additional mutation in PBP3 gene may also have contributed to sulbactam/durlobactam resistance.

In the second isolate, insertion of an additional amino acid at position 374 (-374D) in PBP3 was detected. Unlike A515V and C1546T, this mutation may be associated with high-level resistance to sulbactam and sulbactam/durlobactam because this isolate had sulbactam MIC of >256 MIC mg/L and sulbactam/durlobactam MIC of >64 mg/L. This mutation may have caused a substantial reduction in the affinity of PBP3 for sulbactam.

In our study, sulbactam/durlobactam resistant isolates were also found to encode three silent mutations in PBP3 gene. A silent mutation alters the nucleotide sequence but not the amino acid sequence. The effect of silent mutations has traditionally been considered to be either neutral or nearly neutral. However, recent evidence has demonstrated that they can affect protein levels or conformation [29].

Although we included a small number of isolates in our study, it may be useful to indicate the sulbactam/durlobactam susceptibility rate (91.7%). In a study including 5032 clinical isolates of *A. baumannii-calcoaceticus* complex from 33 countries, sulbactam/durlobactam MIC values for 98.3% of isolates were ≤4 mg/L (susceptible) [8]. In another study including 1722 clinical isolates of *A. baumannii-calcoaceticus* complex from 31 countries, 97.7% of isolates had sulbactam/durlobactam MIC of ≤4 mg/L [11]. However, the collections in these two studies comprised both carbapenem-susceptible and carbapenem-resistant isolates [8, 11].

In the study conducted by Seifert et al., 237 (96.3%) of 246 carbapenem-resistant *A. baumannii* isolates from 37 countries had sulbactam/durlobactam MIC of ≤4 mg/L [30]. The sulbactam/durlobactam susceptibility rate in our set of isolates (91.7%) was similar to that reported by Segatore et al. (92.2%) who evaluated the in vitro activity of the sulbactam/durlobactam against 141 carbapenem-resistant A. baumannii isolates collected from six clinical microbiology laboratories in Italy [31].

Studies which report lower sulbactam/durlobactam susceptibility rates (below 90%) among carbapenem-resistant *A. baumannii* isolates also exist. Petropoulou et al. tested the in vitro activity of sulbactam/durlobactam against 190 carbapenem-resistant *A. baumannii* isolates collected from 11 hospitals throughout Greece and found a susceptibility rate of 87.9% [3].

This study has some limitations. First, we used gradient diffusion method instead of the reference broth microdilution method for the determination of sulbactam and sulbactam/durlobactam MICs. Broth microdilution is known to provide more reliable and accurate results when compared to gradient diffusion method. Second, sulbactam breakpoints for *A. baumannii* have not been formally established by CLSI or EUCAST. As sulbactam is known to be the active component of ampicillin/sulbactam combination against *Acinetobacter* spp., we used the susceptibility breakpoint of 4 mg/L for sulbactam, based on the ampicillin/sulbactam susceptibility breakpoint of 8/4 mg/L established by the CLSI. Although several studies adopted this approach, using a surrogate breakpoint may influence the interpretation of comparative activities of sulbactam and sulbactam/durlobactam against isolates [8, 31, 32]. Third, we could not include detailed clinical information regarding patients because we couldn’t access the complete medical records of patients.

In conclusion, sulbactam/durlobactam demonstrated high activity against carbapenem-resistant *A. baumannii* strains in our study and restored sulbactam susceptibility in ~90% of sulbactam-non-susceptible isolates. Presence of *bla*_PER-1_ in addition to *bla*_OXA−23_ led to an increase in sulbactam MICs but not in sulbactam/durlobactam MICs. Mutations observed in the PBP3 gene of sulbactam/durlobactam resistant isolates may have led to the development of resistance by reducing affinity of PBP3 for sulbactam. Also, lack of the repressor of AdeIJK efflux system in one of the resistant isolates may have contributed sulbactam/durlobactam resistance by causing the overexpression of efflux pumps.

## Data Availability

Sequence data that support the findings of this study have been deposited in GenBank of National Library of Medicine (https://www.ncbi.nlm.nih.gov/) with the accession numbers of PQ382891, PQ382892, and PQ382893.
